# Stereotactic Radiosurgery of Multiple Brain Metastases: A Review of Treatment Techniques

**DOI:** 10.3390/cancers15225404

**Published:** 2023-11-14

**Authors:** Raphael Bodensohn, Sebastian H. Maier, Claus Belka, Giuseppe Minniti, Maximilian Niyazi

**Affiliations:** 1 Department of Radiation Oncology, University Hospital Tübingen, 72076 Tübingen, Germany; raphael.bodensohn@med.uni-tuebingen.de; 2 Center for Neuro-Oncology, Comprehensive Cancer Center Tübingen-Stuttgart, University Hospital Tübingen, 72076 Tübingen, Germany; 3 Department of Radiation Oncology, LMU University Hospital, LMU Munich, 81377 Munich, Germany; sebastian.maier@med.uni-muenchen.de (S.H.M.); claus.belka@med.uni-muenchen.de (C.B.); 4 German Cancer Consortium (DKTK), Partner Site Munich, A Partnership between DKFZ and LMU University Hospital, 81377 Munich, Germany; 5 Bavarian Cancer Research Center (BZKF), Munich, Germany; 6 IRCCS Neuromed, 86077 Pozzilli, Italy; giuseppe.minniti@unisi.it; 7 Department of Radiological Sciences, Oncology and Anatomical Pathology, Sapienza University of Rome, Policlinico Umberto I, 00161 Rome, Italy; 8 German Cancer Consortium (DKTK), Partner Site Tübingen, A Partnership between DKFZ and University Hospital, 72076 Tübingen, Germany

**Keywords:** dynamic conformal arc therapy (DCAT), volumetric arc therapy (VMAT), single isocenter, brain metastases, stereotactic radiosurgery

## Abstract

**Simple Summary:**

In the past year, there have been improvements in cancer treatment, especially for cancers that have spread throughout the body. However, treating tumors that spread to the brain, causing brain metastases, is still a challenge. The brain has extra protection that makes it hard for helpful medications to reach it. That's why local treatments like stereotactic radiosurgery, a precise way of using radiation to treat tumors, are important for addressing brain metastases. Nowadays, even multiple metastases can be treated simultaneously with stereotactic radiosurgery. Different techniques, such as the Gamma Knife that treats metastases one by one, and single-isocenter techniques that can treat many metastases at once using a traditional radiation device called a linear accelerator, are used for this purpose. This article compares the advantages and disadvantages of these treatments by examining other articles published on the topic.

**Abstract:**

The advancement of systemic targeted treatments has led to improvements in the management of metastatic disease, particularly in terms of survival outcomes. However, brain metastases remain less responsive to systemic therapies, underscoring the significance of local interventions for comprehensive disease control. Over the past years, the threshold for treating brain metastases through stereotactic radiosurgery has risen. Yet, as the number of treated metastases increases, treatment complexity and duration also escalate. This trend has made multi-isocenter radiosurgery treatments, such as those with the Gamma Knife, challenging to plan and lengthy for patients. In contrast, single-isocenter approaches employing linear accelerators offer an efficient and expeditious treatment option. This review delves into the literature, comparing different linear-accelerator-based techniques with each other and in relation to dedicated systems, focusing on dosimetric considerations and feasibility.

## 1. Introduction

Treatment of metastatic cancer diseases have partly evolved from mostly palliative interventions with whole-brain radiotherapy (WBRT) to semicurative strategies. This shift can be largely attributed to the advancement of checkpoint inhibitors, such as the cytotoxic T-lymphocyte-associated Protein 4 (CTLA4) inhibitor ipilimumab as well as “Programmed cell death protein” (PD-1) inhibitors like nivolumab and pembrolizumab [1,2,3]. With improved systemic control, local approaches, in particular radiation therapy, became indispensable for refractory sites or areas less responsive to systemic treatment such as the brain [4,5]. Until just a few decades ago, localized treatment of brain metastases (BM) through surgery or stereotactic radiosurgery (SRS) was predominantly reserved for patients with a limited number of BM, typically ranging from one to three or four metastases depending on the literature [6,7]. Recent technological advancements, however, have now made it possible to treat multiple metastases with SRS as well [8]. The recently published STEREOBRAIN study, which prospectively compared SRS to a historical WBRT cohort for patients with 4–10 BM, demonstrated a trend toward improved survival for patients treated with SRS [9]. The latest “European society of medical oncology”– “European association of neurooncoology” (ESMO-EANO) guideline on BM treatment recommends that “SRS may be considered for patients with a higher number of BM (5–10) with a cumulative tumour volume < 15 mL” [10]. Considering that SRS for multiple metastases is a complex procedure and can be accomplished through various methods, this review explores the landscape of SRS for multiple metastases, with a focus on the key treatment techniques.

## 2. Number of Metastases: How Many Are Too Many?

While there are previous studies that investigated SRS for multiple metastases [11,12,13], it is widely recognized that the prospective observational study conducted by Yamamoto et al., published in 2014, marked a significant milestone and is often regarded as the starting point for the practice of SRS for multiple metastases [8]. The study prospectively enrolled 1194 patients who underwent SRS for 1–10 metastases. Among them, 455 patients had a solitary metastasis, 531 patients had 2–4 metastases, and 208 patients had 5–10 metastases. The overall survival and adverse effects observed in the group that was treated with SRS for 5–10 metastases did not exhibit significant differences from the group with 2–4 metastases, indicating that SRS for 5–10 metastases is noninferior to SRS for 2–4 metastases. A follow-up study from 2017 confirmed these outcomes, demonstrating no inferiority in terms of cognitive toxicity as well [14]. Additional cohort studies conducted by the same researchers revealed that treating patients with SRS for up to 15 or even 20 metastases was feasible in carefully selected cases [15,16,17]. It appears that a definitive numerical limit for SRS has yet to be established. Further investigations propose that the cumulative tumor volume of the metastases, rather than solely the count, provides a more accurate indicator of the feasibility of SRS [18,19]. As outlined in the mentioned ESMO-EANO guideline, a tumor volume of 15 mL is typically considered the maximum volume that can be safely treated with SRS [10]. However, it is essential to note that this figure is primarily derived from studies employing the Gamma Knife (GK) technique, such as the one conducted by Yamamoto et al. in 2014. With GK, metastases are usually irradiated without a “planning target volume” (PTV) margin, which results in smaller irradiation volumes, while in LINAC-based plans, where margins of 1–2 mm are commonly employed, the irradiation volumes could be relatively larger.

Regarding SRS treatment, most guidelines indicate that prescription doses may vary within the range of 15–24 Gy, depending on size and location [6,7,10]. Based on clinical experience, lower doses, with a maximum of 20 Gy, appear to be sufficient for local tumor control [9,20,21,22,23,24]. An earlier review by Shiau et al. in 1997 indicated that doses of 18 Gy or higher already exhibit effective local tumor control [25]. In the context of SRS for multiple metastases, each treated lesion has an impact on the individual lesion dose of the others. To mitigate this influence, Sahgal et al. suggest reducing the prescribed dose to each target by 1–2 Gy [26] for multitarget radiotherapy. Although this suggestion is also used for SI techniques [9,20], it is unclear whether the impact is similar.

## 3. Planning Quality Indicators

Before presenting studies that compare various technical approaches, it is essential to introduce a few commonly used parameters for evaluating SRS plans. It is, however, important to note that these parameters were originally meant for SRS of single metastases. Interpretation for multiple metastases is therefore not that trivial, as several targets contribute to the total dose. Mostly, these parameters are looked at for each lesion separately in a smaller region around the target.

To assess plan conformity, Shaw et al. introduced the RTOG conformity index (CI), which represents the ratio of the prescription isodose volume (PIV) to the target volume (TV) [27]. Ideally, the CI should be ≤ 1.2:

$$CI=\frac{PIV}{TV}$$

Due to the possibility of the CI indicating good conformity even when the PIV and CIV do not cover each other, Paddick et al. introduced a new conformity index (PI) that addresses this limitation [28]. This index is calculated as the squared volume of the target covered by the PIV (TV_PIV_) divided by the product of the TV and the PIV. The outcome provides a percentage representation of conformity:

$$PI=\frac{{TV}_{PIV}^{2}}{TV\times PIV}$$

To quantify the steepness of the dose gradient, the gradient index (GI) is frequently employed [29]. It is calculated as the ratio between the isodose volume receiving half of the prescribed dose (PIV_half_) and the PIV. Ideally, the GI should be around 2–3:

$$GI=\frac{{PIV}_{half}}{PIV}$$

Given that radiation necrosis is a crucial adverse event following SRS, many studies also analyze the risk associated with each individual treatment plan. The V10 and V12 values (representing the volume covered by 10 Gy and12 Gy, respectively) of the healthy brain tissue (usually calculated as brain volume minus the gross tumor volume (GTV)) are widely utilized as indicators for assessing the risk of radiation necrosis [30,31]. In cases involving plans with multiple PTVs, the assessment is typically limited to the brain area surrounding each metastasis. For optimal outcomes, the V10 and V12 values should ideally be ≤10 cc and ≤8 cc, respectively [30,31,32].

In addition, certain studies also examined the volume of healthy brain tissue encompassed by very low isodose levels, such as V3 and V5, in order to evaluate the extent of dose spread within the treatment plan.

## 4. Image Guidance in Single-Isocenter Techniques

Traditionally, SRS was performed using a stereotactic frame affixed directly to the patient’s skull. Subsequently, frameless methods utilizing a thermoplastic mask gained prominence, providing a noninvasive yet somewhat less precise approach to patient immobilization [33,34]. Due to the fixed arrangement of cobalt sources, GK benefits from having to accommodate fewer potential positioning errors, often requiring minimal to no safety margins. In non-coplanar LINAC radiosurgery, both the couch and collimator movement present potential sources for positioning errors. In the case of single-isocenter (SI) approaches, rotational errors hold particular significance. This is especially critical since some lesions are located at a distance from the isocenter, rendering them more vulnerable to shifts or alterations in positioning. Cone-beam computed tomography (CBCT) is adept at identifying translational and rotational errors, allowing for their subsequent correction using six-dimensional couch correction systems. While the corrections usually suffice for coplanar techniques, CBCT cannot be used in swapped-out couch positions used for non-coplanar approaches. Thus, errors occurring during couch movement cannot be corrected. To compensate for the rotational error, some authors add a higher margin to lesions distant from the isocenter [35,36,37].

An alternative option involves control systems capable of monitoring positions across all couch orientations. A specialized X-ray control system exhibited positioning accuracy comparable to frame-based approaches [38]. Much like CBCT, it interfaces with six-dimensional couch correction systems, and its positioning accuracy is similar [36,37,39]. However, it holds the distinct advantage of monitoring intrafractional motion during the course of treatment [40,41]. As precise planning for stereotactic radiosurgery often requires non-coplanar angles, X-ray control systems offer a distinct advantage toward CBCT: X-ray can also verify the patient’s position in a couch angle that does not allow CBCT [40]. Through this, potential positioning errors that occur during couch movements can be detected and corrected accordingly. Furthermore, additional optical surface tracking systems can contribute to diminished translational errors of 0.3 mm and rotational errors of 0.4° [42,43]. As outlined by Roper et al.’s calculations, a rotational error of 0.5° displayed D95 and V95 coverage exceeding 95% across all cases [35]. Tracking the patient during treatment seems to be important especially for SRS due to its long treatment time, as intrafractional movement increases for long treatments [44]. Da Silva Mendes et al. found differences between optical/thermal surface guidance and X-ray imaging of a maximum of 0.02 mm for 14 patients, making surface guidance an effective tool for real time intrafractional tracking [40].

To summarize, SI-LINAC SRS is susceptible to rotational errors when compared to multi-isocenter methods, as these errors directly impact coverage, particularly for lesions positioned far from the isocenter. Nevertheless, advancements in image guidance systems featuring X-ray control systems and OST have the potential to effectively counteract rotational errors. Continuous improvement in image guidance, however, is vital if higher numbers of BM are to be treated in the future.

## 5. Technical Approaches and Different Technological Solutions

GK, developed by the Swedish neurosurgeon Lars Leksell in the 1950s, is the first device employed in radiosurgery [45,46]. This system currently uses 192 ^60^Co-sources arranged in a half-sphere, all converging at a single isocenter, facilitating highly precise irradiation of small lesions. Due to its design, which permits the irradiation of only one distinct lesion at a time, the process of performing GK radiosurgery for multiple lesions becomes progressively time-consuming as the number of lesions increases [47,48]. Later in the 1980s, LINAC-based SRS was introduced, and since then, it has steadily advanced to achieve planning quality comparable to that of GK [48,49,50,51].

In multi-isocenter approaches utilizing LINAC or GK, the amount of healthy brain tissue affected expands with each additional target due to the interplay between target plans [52]. Conversely, a more efficient strategy is the single-isocenter (SI) approach with a linear accelerator (LINAC). In this method, a single rotational center is employed to simultaneously treat multiple metastases. The treatment planning system calculates optimal arcs to achieve a good lesion coverage and at the same time minimize the impact on healthy brain tissue [53,54,55,56,57]. Also, dedicated radiosurgery systems are developing toward SI approaches [58]. Several studies conducted comparisons between SI techniques and GK as well as between different SI techniques. In the following sections, we will present these studies and outline the dosimetrics of each technique. An overview of all the discussed studies is provided in Table 1.

Thomas et al. conducted a comparative study between GK and SI-VMAT in 28 cases with a total of 113 lesions [48]. The results indicated that the SI-VMAT plans exhibited better conformality for each target in terms of both the CI and the PI when compared to the GK plans. However, there was no discernible difference in the cumulative V12 value. The median beam-on time for SI-VMAT and GK plans was 45.1 min (range: 17.5–121.2) and 125 min (range: 60–310), respectively [48]. Liu et al. performed a similar comparison involving six patients, each with three to four lesions, and found that SI-VMAT plans had a smaller conformity index (CI) but a larger gradient index (GI) compared to GK plans [59]. Similar to Thomas et al., they observed comparable V12 values between the two techniques. Additionally, the steeper dose drop-offs associated with GK led to smaller low-dose isodoses (V3) of the healthy brain tissue in comparison to SI-VMAT [59]. Potrebko et al. conducted an analysis involving 12 patients with a total of 103 metastases and a median of 8 (range: 7–14) metastases per patient [24]. They compared the treatment patients received with non-coplanar VMAT plans utilizing 6-MV and 10-MV flattening filter-free (FFF) modes to GK. Differing from the previously mentioned studies, Potrebko et al. found that the lesion-specific V12 value was significantly lower for GK plans in comparison to SI-VMAT plans. However, conformality was similarly superior with the SI-VMAT plans compared to GK. In summary, SI-VMAT demonstrated better (or at least comparable) conformality in most studies and offered shorter treatment times, while GK exhibited a more favorable dose gradient. Furthermore, in one study, GK displayed superior V12 values to some extent [24].

Regarding the direct comparison between GK and SI-DCAT, there are hardly any publications. Chea et al. investigated 20 patients with a total of 95 metastases, with each patient having three to nine lesions [21]. They observed that conformity, measured with PI, was marginally better for SI-DCAT plans. This improvement became statistically significant for small lesions (<1 cc) and lesions with a high degree of sphericity (>0.78). The dose gradient, assessed by the gradient index (GI), was significantly steeper in GK. However, there was no noteworthy difference in V12 values between the two types of plans. The authors concluded that GK and SI-DCAT plans demonstrated comparable planning qualities, but GK exhibited higher healthy brain tissue sparing [21]. Another study by Ruggieri et al. also compared GK to SI-DCAT but also included multifraction RT. For this reason we did not include it in this comparison [60].

**Table 1:** An overview of the mentioned studies comparing the treatment techniques. Abbreviations: PTV—planning target volume; GTV—gross tumor volume; CI—conformity index; PI—Paddick conformity index.

Study	Details		Gamma Knife	SI-VMAT	SI-DCAT	*p*-Value
Liu et al. [59]	6 patients, 19 lesions. Median 3 (3–4)/patient. Median total PTV 3.6 cc (1.2–11.1). Median PTV 0.5 (1.1–10.5). GTV to PTV margin not known.	Mean CI (target)	1.5 ± 0.2	1.2 ± 0.1		<0.001
		Mean GI (target)	3.7 ± 1.0	4.8 ± 1.5		<0.01
		Mean V12 (cc) (target)	3.1 ± 2.2	2.7 ± 1.4		0.58
		Mean V12 (cc) (total)	10.9 ± 7.2	9.7 ± 5.1		0.63
		Mean V6 (cc)	36.9 ± 16.9	36.3 ± 14.7		0.96
		Mean V4.5 (cc)	86.7 ± 29.8	99 ± 27.3		0.15
		Mean V3 (cc)	160.8 ± 55.7	224 ± 53		0.1
		Mean beam-on time (min)	71.6 ± 15.9	6.4 ± 0.8		<0.01
Thomas et al. [48]	28 patients, 113 lesions. Median 3 (2–9)/patient. Median total PTV 3.7 cc (0.2–19.6). Median PTV 0.1 cc (0.003–15.0). GTV to PTV margin not known.	Median CI (total)	1.7 (1.3–7.4)	1.1 (1.0–1.7)		<0.0001
		Median PI (total)	0.6 (0.3–0.8)	0.9 (0.6–0.9)		<0.0001
		Median CI (target)	1.9 (1.2–6.1)	1.3 (1.0–4.3)		<0.0001
		Median PI (target)	0.5 (0.2–0.8)	0.8 (0.2–1.0)		<0.0001
		Median beam-on time (min)	45.1 (17.5–121.3)			
Potrebko et al. [24]	12 patients, 103 lesions. Median 8 (7–14)/patient. Mean GTV 1.16 cc (0.01–19.9). Prescription dose 15–21 Gy. GTV to PTV margin 0 mm.	Mean CI (target)	2.5 ± 1.6	1.6 ± 0.8 (6 MV) 1.7 ± 0.9 (10 MV)		<0.001 (6 MV) <0.001 (10 MV)
		Mean V12 (cc) (total)	24 ± 21	25 ± 17 (6 MV) 26 ± 18 (10 MV)		0.835 (6 MV) 0.705 (10 MV)
		Mean V12 (cc) (target)	2.8 ± 6.1	3.0 ± 5.2 (6 MV) 3.1 ± 5.4 (10 MV)		0.003 (6 MV) <0.001 (10 MV)
		Mean V6 (cc) (total)	81.1 ± 72.9	143.7 ± 81.1 (6 MV) 167.5 ± 87.5 (10 MV)		0.09 (6 MV) 0.01 (10 MV)
		Mean V3 (cc) (total)	323.0 ± 294.8	880.1 ± 369.1 (6 MV) 937.9 ± 361.9 (10 MV)		0.005 (6 MV) 0.001 (10 MV)
		Median beam-on time (min)	147.6 ± 49.3	10.8 ± 2.1 (6 MV) 6.4 ± 1.2 (10 MV)		0.01 (6 MV) <0.001 (10 MV)
Chea et al. [21]	20 patients, 95 lesions. 3–9/patient. Median GTV 0.3 cc (0.02–9.6). Prescription dose 20 Gy. GTV to PTV margin 0 mm.	Mean PI (target)	0.5 ± 0.2		0.6 ± 0.1	0.07
		Mean GI (target)	3.2 ± 0.6		4.1 ± 1.1	<0.001
		Mean V12 (cc) (target)	1.7 ± 2.3		1.9 ± 2.6	0.013
		Mean beam-on time (min)	169 ± 48		94 ± 26	<0.001
Raza et al. [22]	36 patients, 367 lesions. Median 9 (2–25)/patient. Median total GTV 1.33 cc (0.2–4.7). Median GTV 0.16 cc (0.1–2.1). Median total PTV 3.4 cc (0.6–9.4). Median PTV 0.37 (0.2–1.2). Median prescription dose 20 Gy (18–20). GTV to PTV margin 1 mm.	Median PI (target)		0.6 (0.5–0.9)	0.8 (0.5–0.9)	0.005
		Median GI (target)		5.6 (3.6–8.4)	4.5 (3.5–7.1)	<0.0001
		Median V12 (cc) (total)		18.5 (2.2–62.3)	13.6 (1.9–45.9)	<0.0001
		Median V12 (cc) (total) (1–10 lesions)		8.2 (2.2–26.4)	6.2 (1.87–18.1)	0.001
		Median V12 (cc) (total) (10–25 lesions)		38.7 (21.9–62.3)	22.7 (12.2–45.9)	0.001
		Median V10 (cc) (total)		27.9 (2.9–100.6)	17.4 (2.6–74.2)	<0.0001
		Median V8 (cc) (total)		46 (4.6–184)	26.6 (3.7–157)	<0.0001
		Median V5 (cc) (total)		142.3 (11.3–707.8)	73.2 (8.18–701.8)	<0.0001
Hofmaier et al. [61]	20 patients, 66 lesions. Median 3 (2–6). Median PTV 0.8 cc (0.1–11.9). Prescription dose 15–20 Gy. GTV to PTV margin 1 mm.	Median PI (target)		0.73 (0.38–0.88)	0.75 (0.58–0.89)	<0.05
		Median GI (target)		7.17 (3.35–33.0)	5.99 (3.5–15.73)	<0.05
		Median V12 (cc) (target)		3.1 (0.5–13.9)	2.1 (0.1–13.1)	<0.05
		Median V10 (cc) (target)		4.9 (1.0–19.9)	3.2 (0.4–19.3)	<0.05
		Median V8 (cc) (total)		26.6 (10.6–86.4)	17.4 (6.3–52.6)	<0.05
		Median V5 (cc) (total)		69.1 (26.0–273.6)	33.7 (13.0–120.5)	<0.05
		Median V4 (cc) (total)		123.1 (37.5–418.6)	45.6 (18.5–215.9)	<0.05
Gevaert et al. [23]	10 patients, 40 lesions. Median 3 (1–8)/patient. Mean GTV 3.15 cc (0.1–24.6). Mean total PTV 10.60 (0.6–28.7). Prescription dose 20 Gy. GTV to PTV margin ≤ 1 mm (variable).	Mean PI (target)		0.7 ± 0.2	0.7 ± 0.1	
		Mean GI (target)		7.1 ± 3.1	3.9 ± 1.4	<0.05
		Mean V12 (cc) (total)		46.3 ± 35.9	36.3 ± 27.1	<0.05
		Mean V10 (cc) (total)		67.9 ± 55.9	48.5 ± 35.9	<0.05
		Mean V5 (cc) (total)		266.7 ± 216.7	161.6 ± 143.6	
Liu et al. [62]	30 patients, 217 lesions. 7.5 (4–10)/patient. Median total PTV 7.1 cc. Median PTV 0.4 cc. Median prescription dose 18 Gy (14–24). GTV to PTV margin ≤ 1 mm (variable).	Median CI (target)		1.2 ± 0.3	1.4 ± 0.3	<0.0001
		Median PI (target)		0.8 ± 0.2	0.7 ± 0.1	<0.0001
		Median V12 (cc) (total)		19.2	23.7	<0.0001
		Median V8 (cc) (total)		44.1	53.6	0.024
		Median V5 (cc) (total)		142.8	141.4	0.009
		Dmean (Gy)		2.8	2.6	<0.0001

Several other studies conducted comparisons between SI-VMAT and SI-DCAT techniques. Gevaert et al. compared both techniques against each other and with multi-isocenter approaches in 10 patients with 40 lesions and median 3 (1–8) lesions per patient [23]. Both SI-LINAC techniques outperformed multi-isocenter SRS. Moreover, SI-DCAT demonstrated superiority over SI-VMAT in terms of dose gradient, V12, and V5 values. However, the conformity of both SI plans was similarly favorable. Raza et al. investigated 36 patients encompassing a total of 367 metastases, with each patient having 2–25 lesions [22]. Their study demonstrated the superiority of SI-DCAT, particularly for cases involving higher numbers of metastases. They observed significant improvements in PI, GI, V12, and V5 values with SI-DCAT plans compared to SI-VMAT plans [22]. In contrast, another study conducted by Liu et al., which involved 30 patients with a total of 217 metastases and 4–10 lesions per patient, reported opposite results: They found that SI-VMAT plans exhibited improved conformity and V12 values compared to SI-DCAT plans [62]. The only parameter in which SI-DCAT plans outperformed SI-VMAT plans was in V5 values. The disparity between these two studies could potentially be attributed to the size and number of the metastases involved. In the study by Raza et al., where the median number of lesions per patient was nine and the median total PTV was 3.4 cc, SI-DCAT plans demonstrated superiority. Conversely, in the study by Liu et al., with a median of 7.5 lesions per patient and a median total PTV of 7.05 cc, SI-VMAT plans exhibited better conformity and V12 values. This discrepancy might suggest that SI-DCAT performs better for numerous small targets, while SI-VMAT is more effective for fewer but larger targets. This pattern is particularly noticeable in the Raza et al. study, where the advantage of SI-DCAT increased for a higher number of metastases (10–25) [22]. Vergalasova et al. conducted a study comparing 16 patients with a total of 112 metastases, comparing GK, SI-DCAT, and three SI-VMAT methods (as only the differences and not the absolute values of the parameters are given, this study is not included in Table 1). Their conclusion was that SI-VMAT planned with HyperArc^®^ exhibited superiority over the other systems, while GK demonstrated the best dose gradient. It is noteworthy that, similar to the cohort of Liu et al., the median PTV in the study by Vergalasova et al. was relatively large (12.6 cc, range: 1.8–23.5 cc), and the median number of lesions (7, range: 4–10) was smaller than that in the Raza et al. study. However, the aforementioned study by Gevaert et al. had a large mean total PTV of 10.6 cc (range: 0.6–28.7) and a small median number of lesions of three (range: 1–8) per patient but still showed superior results for SI-DCAT. It remains unclear if the number and size determine which technique is better suited. A study by Hofmaier et al. reported comparable PI for SI-VMAT and SI-DCAT and superiority of SI-DCAT in all other investigated parameters (GI, V12, V10, V8, V5, V4) but found that SI-VMAT could be superior for nonspherical target volumes [61]. It would be interesting to know the sphericity of the lesions analyzed in the other studies: a larger lesion might tend to be more irregular, explaining the results in Liu et al. and Vergalasova et al.

**Figure 1:**
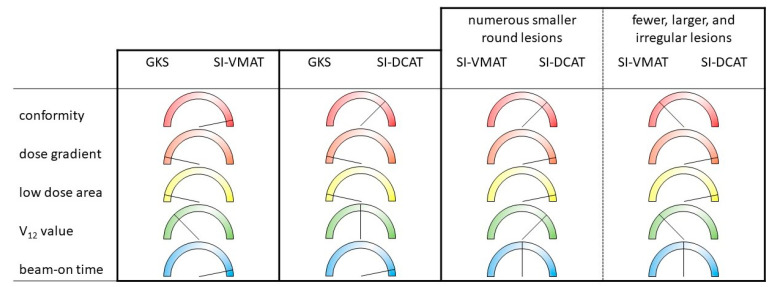
A relative comparison between the different treatment techniques; GK—Gamma Knife; SI-DCAT—single-isocenter dynamic arc therapy; SI-VMAT—single-isocenter volumetric arc therapy.

In summary, GK generally shows a steeper dose gradient and tends to have lower V12 values compared to SI-LINAC methods. Conversely, both SI-VMAT and SI-DCAT techniques exhibit notably shorter treatment times and improved conformity. However, when comparing SI-VMAT and SI-DCAT, conflicting results in terms of V10/V12, GI, and PI are observed. Nonetheless, it appears that SI-DCAT tends to result in lower values for low-dose areas (V5). Figure 1 offers an overview highlighting the distinctions between the various treatment techniques discussed in this review with no intention to provide absolute relations.

## 6. Conclusions

In conclusion, upon a comprehensive literature review, SI-LINAC systems emerge as the preferable choice for the majority of patients requiring treatment for multiple metastases, as they offer a time-efficient alternative to GK while maintaining comparable dosimetric characteristics [21,22,24,48,59,62,63]. Moreover, it remains uncertain whether the presumed advantages of GK, such as steeper dose gradients and lower healthy brain exposure, truly translate to reduced toxicity and radionecrosis risk. A study by Sebastian et al. compared GK and LINAC SRS, focusing specifically on radionecrosis rates [50]. Surprisingly, the study revealed that GK exhibited a higher rate of radionecrosis in comparison to LINAC SRS despite both treatments showing similar survival rates. The authors put forward a hypothesis suggesting that the elevated delivered dose to the center of the tumor, which is a characteristic feature of GK dose prescription, might be accountable for the heightened rates of radionecrosis. In the end, the endeavors to minimize parameters conventionally linked to radionecrosis, such as V12, may not hold as much significance as previously assumed. There could be a necessity for alternative parameters in estimating the risk of radionecrosis, possibly involving calculations based on the equivalent uniform dose (EUD) to refine treatment techniques accordingly [64,65].

Given the potential benefits of SRS for multiple metastases when compared to conventional whole-brain radiotherapy, it is crucial to prioritize the optimization of technical approaches [9]. For SI-LINAC, it is advisable to consider the integration of image-guided and surface-guided systems providing intrafractional motion tracking, as they can significantly contribute to the reduction in translational and rotational errors during multiarc non-coplanar treatments [20,40,42,43]. In the comparisons between SI-DCAT and SI-VMAT, conflicting outcomes were observed. However, it appears that SI-DCAT is more advantageous for numerous smaller round lesions, while SI-VMAT is preferable for fewer, larger, and irregular lesions. Clinically, both techniques were shown to be feasible [9,17,20,30,33,66].

In conclusion, the future of medical treatment techniques in the field of SRS holds great promise. It is highly likely that advances in intrafraction control will continue to evolve, reducing the required safety margins and enhancing treatment precision. Furthermore, a notable area of potential improvement lies in streamlining the planning process, thereby minimizing the time gap between planning images (such as CT and MRI) and SRS and thus diminishing uncertainties due to potential changes in treatment volumes occurring between imaging and treatment [67]. Next to the advancement of technical approaches, it is important to further establish the exact role and indication for SRS. As shown in several studies, SRS is very effective when combined with targeted therapy or immunotherapy [4,68,69,70]. However, the sequence in which SRS and systemic treatment should be applied first still remains unclear and is a frequently discussed topic [5,71,72,73,74,75]. Also, the question of which fractionation should be used for best results still needs further discussion [76]. In total, several technical and clinical issues still require further clarification and exploration in the future.
